# Variability of interobserver interpretation of selected helminth ova in the development of a training image set

**DOI:** 10.1093/inthealth/ihae085

**Published:** 2025-01-09

**Authors:** Rupert Stephen Charles S Chua, Kiersten A Henderson, Lorenzo Maria C de Guzman, Vicki Foss, Nathaniel Schub, Cameron Bell, John Robert C Medina, Taggart G Siao, Myra S Mistica, Maria Luz B Belleza, Marie Cris R Modequillo, Nadine Joyce C Torres, Vicente Y Belizario

**Affiliations:** Neglected Tropical Diseases Study Group, National Institutes of Health, University of the Philippines Manila, Manila, Philippines; Parasite ID, Corp., Seattle, WA, USA; Neglected Tropical Diseases Study Group, National Institutes of Health, University of the Philippines Manila, Manila, Philippines; Parasite ID, Corp., Seattle, WA, USA; Parasite ID, Corp., Seattle, WA, USA; Parasite ID, Corp., Seattle, WA, USA; Institute of Clinical Epidemiology, National Institutes of Health, University of the Philippines, Manila, Manila, Philippines; Neglected Tropical Diseases Study Group, National Institutes of Health, University of the Philippines Manila, Manila, Philippines; Department of Parasitology, College of Public Health, University of the Philippines Manila, Manila, Philippines; Department of Parasitology, College of Public Health, University of the Philippines Manila, Manila, Philippines; Department of Health Davao Center for Health Development, Davao City, Philippines; Department of Health Caraga Center for Health Development, Butuan City, Philippines; Neglected Tropical Diseases Study Group, National Institutes of Health, University of the Philippines Manila, Manila, Philippines; Department of Parasitology, College of Public Health, University of the Philippines Manila, Manila, Philippines

**Keywords:** artificial intelligence, interobserver variability, schistosomiasis, soil-transmitted helminths

## Abstract

**Background:**

Diagnosis of soil-transmitted helminthiasis and schistosomiasis for surveillance relies on microscopic detection of ova in Kato–Katz (KK) prepared slides. Artificial intelligence (AI)-based platforms for parasitic eggs may be developed using a robust image set with defined labels by reference microscopists. This study aimed to determine interobserver variability among reference microscopists in identifying parasite ova.

**Methods:**

Images of parasite ova taken from KK prepared slides were labelled according to species by two reference microscopists (M1 and M2). A third reference microscopist (M3) labelled images when the first two did not agree. Frequency, percent agreement, κ statistics and variability score (VS) were generated for analysis.

**Results:**

M1 and M2 agreed on 89.24% of the labelled images (κ=0.86, p<0.001). M3 had agreement with M1 and M2 (κ=0.30, p<0.001 and κ=0.28, p<0.001), resolving 89.29% of disagreement between them. The labelling of *Schistosoma japonicum* had the highest VS (κ=0.487, p=0.101) among the targeted ova. Reference microscopists were able to reliably reach consensus in 99.0% of the dataset.

**Conclusions:**

Training AI using this image set may provide more objective and reliable readings compared with that of reference microscopists.

## Introduction

Neglected tropical diseases (NTDs) are infectious diseases of poverty that impose human, social and economic burden on >1 billion people worldwide.^1^ Among the parasitic NTDs are soil-transmitted helminthiasis (STH), which infects approximately 1.5 billion people, and schistosomiasis (SCH), which affects almost 240 million people globally.^2,3^ The Philippines is still considered endemic for all STH infections and SCH is endemic in 190 municipalities in 28 provinces despite more than a decade of control program implementation.^4^

The Kato–Katz (KK) technique remains the recommended diagnostic standard to detect and quantify the intensity of STH and SCH for surveillance activities.^1^ However, the low sensitivity of the test for both STH and SCH ova, in light-intensity infections,^5^ and the low accuracy of ova speciation remain challenges.^6^ Development of a KK-based technology to detect parasite ova using artificial intelligence (AI), in consideration of resource-limited settings, supports the World Health Organization (WHO) 2030 STH roadmap.^7^ AI in microscopy can help provide standardized results on a par with experienced microscopists, provided that a reliable training image set is available.

To be able to build AI technologies using a machine learning algorithm, a fully labelled image dataset is a valuable tool for reproducible results and is used to train the machine learning algorithm,^8^ yet currently there is no readily available standardized library of labelled images of soil-transmitted helminths and *Schistosoma japonicum* ova that can be used as a training dataset for machine learning.

Interpretation of images from more than one reference microscopist is needed to provide a reliably labelled training image set since microscopy relies heavily on morphology and different readers can have varying professional opinions on certain images.^9^ To address the variability of results among observers to provide a reliable training image set, the interobserver variability among three reference microscopists was observed.

Interobserver variability in reading of microscopic slides has been explored in clinical histopathology using both traditional glass slide reading and digital pathology reading and generally show high interobserver agreement between pathologists.^10^ In clinical parasitology, where microscopy remains as the primary method of diagnosis,^11^ similar studies on interobserver variability are limited and traditional glass slide reading shows poorer agreement between microscopists with different levels of experience.^12^

This study aimed to determine interobserver variability and patterns of variable interpretation between reference microscopists in labelling images of parasite ova processed using the KK technique to develop a training image set for use in the development of machine learning–based algorithms for AI technologies to improve the diagnosis of STH and SCH.

## Methods

### Study site

Image collection was conducted in selected municipalities of provinces known to be endemic for STH and SCH, with low mass drug administration (MDA) coverage defined as <75%,^1^ based on data from local government units (LGUs). The study sites identified were Bunawan and Trento in Agusan del Sur and Kapalong in Davao del Norte, Philippines.

### Data collection

A community-based stool collection in coordination with the LGUs of the selected study sites was done. There was a total of 1159 unique samples submitted during the study across three study sites in two regions. Stool samples were processed within 24 h of collection using the KK technique following the bench aids provided by the WHO.^11^ Preliminary screening was conducted by trained field microscopists.

Microscope slides created from the samples were scanned using the 10× objective magnification (numerical aperture [NA]=0.25) of an Olympus CX21 microscope and were shifted to 40× magnification (NA=0.65) for image capturing once objects of interest were identified. Images were captured using the main camera (48MP [f/2.0]) of a Samsung Galaxy A51 smartphone^13^ through a universal mobile phone holder clamp (eyepiece diameter range: 2.8–4.7 cm, phone slot range: 5.5–8.5 cm) and uploaded to a secure online database. Each sample screened as positive for STH or SCH provided the objects of interest for labelling.

A box was drawn around the identified objects of interest in each image and two reference microscopists (M1 and M2) were tasked with labelling the boxed objects using the Azure AI Machine Learning Studio Data Labeling Platform^14^ based on parasite egg species. Artifacts were labelled as ‘negative’ and identified ova were labelled ‘*Ascaris lumbricoides*–fertilized’, ‘*Ascaris lumbricoides*–unfertilized’, ‘hookworm’, ‘*Trichuris trichiura*’, ‘*Schistosoma japonicum*’ or ‘other egg type or organism’.

Any egg-looking object matching the description of *Ascaris, Trichuris*, hookworm or *S. japonicum* ova were included as an object of interest. Any other parasite ova identified were also boxed as objects of interest but were labelled as other egg type or microorganism. Fields of view with egg-looking objects (artifacts) to be boxed were counted as negative.

In case of disagreement between M1 and M2, a third reference microscopist (M3) was assigned as an arbiter to re-examine the objects and provide a final label. All reference microscopists labelled the images independently and were blinded to the results of the other readers. The reference microscopists were all specialists and trainers for parasite microscopy training with at least 10 y of experience.

### Data processing and analysis

The frequency of agreement and disagreement between reference microscopists, frequency of mismatched label pairs between the first and second reference microscopists and frequency of mismatched label groups among all three microscopists were calculated per species using an Excel spreadsheet (Microsoft, Redmond, WA, USA). The percent agreement was calculated by dividing the number of agreements by the total number of objects. Multivariable κ statistics calculated using SPSS Statistics 25 (IBM, Armonk, NY, USA) were used to determine interobserver agreement between reference microscopists.^15^ A variability score was calculated using equation (1), where a score closer to a whole number represents higher variability. To determine patterns of variable interpretation, a chord diagram was created using RStudio version 2023.12.1 Build 402 (Posit Software, Boston, MA, USA).


(1) \begin{eqnarray*}
\textit{Variability}{\mathrm{\, }}\textit{Score} &=& \frac{{No.{\mathrm{\, }}of{\mathrm{\, }}\textit{disagreements}{\mathrm{\, }}per{\mathrm{\, }}\textit{specimen}{\mathrm{\, }}\,\textit{type}}}{{No.{\mathrm{ \, }}of{\mathrm{\, }}\textit{agreed}{\mathrm{\, }}\textit{final}{\mathrm{\, }}\textit{readings}{\mathrm{\, }}per{\mathrm{\, }}\textit{specimen}{\mathrm{\, }}\,\textit{type}}}\nonumber\\
\end{eqnarray*}

## Results

### Preliminary screening

Of the 1159 unique samples submitted in the preliminary screening, 331 (28.56%), 44 (3.80%), 115 (9.92%) and 71 (6.13%) were positive for *A. lumbricoides, T. trichiura*, hookworm and *S. japonicum*, respectively (Table 1). From these samples, a total of 18 435 fields of view were captured as individual images from which objects of interest were identified.

**Table 1.** Post-screening prevalence of STH and SCH in selected municipalities in the Philippines, October–November 2021

Region	Municipality	Participants examined, n	Positive, n (%)				
			Any STH	*Ascaris lumbricoides*	*Trichuris trichiura*	Hookworm	*Schistosoma japonicum*
Caraga	Bunawan	190	34 (17.89)	19 (10.00)	0	17 (8.95)	42 (22.11)
	Trento	234	20 (8.55)	10 (4.27)	1 (0.43)	11 (4.70)	21 (8.97)
Davao	Kapalong	735	358 (48.71)	302 (41.09)	43 (5.85)	87 (11.84)	8 (1.09)
Total		1159	412 (35.55)	331 (28.56)	44 (3.80)	115 (9.92)	71 (6.13)

### Final object readings

**Figure 1.**
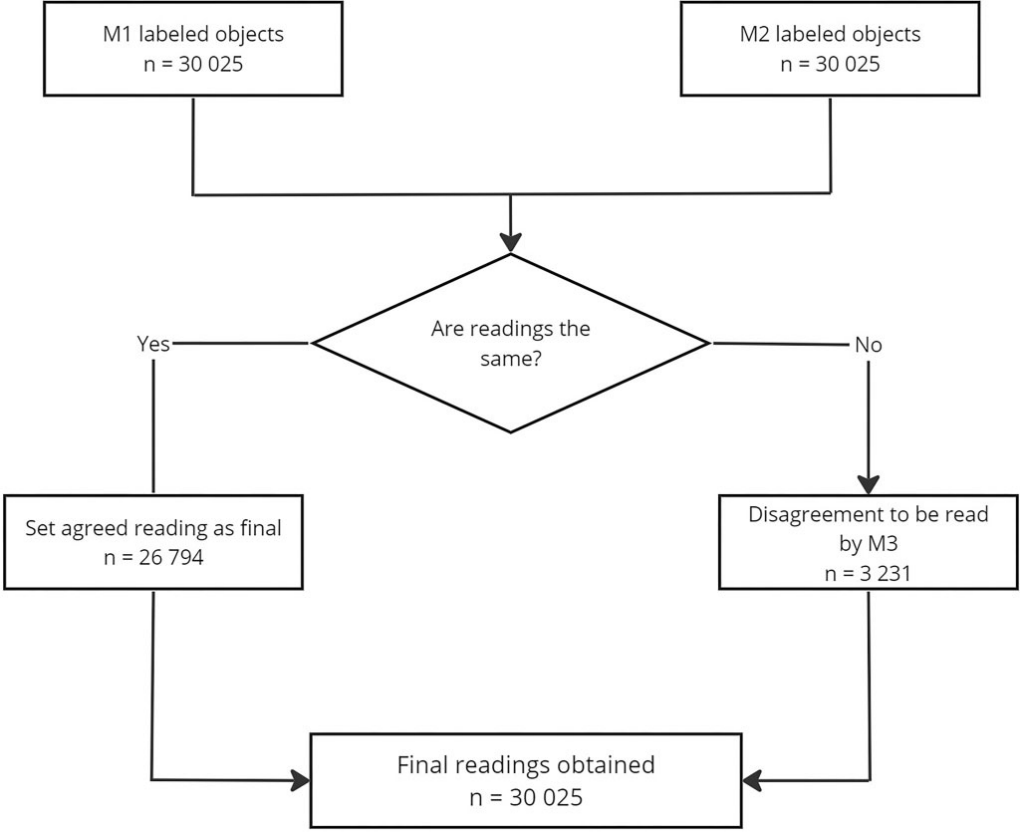
Schematic diagram on obtaining a final reading. M1 and M2 independently labelled 30 025 objects of interest. Agreements are taken as the final object reading while disagreements were refereed by M3 to obtain a final object reading.

A total of 30 025 individual objects were identified from each field of view captured as images containing parasite ova or artifacts resembling parasite ova. M1 and M2 agreed on labels for 26 794/30 025 (89.24%) objects and these were used as the final reading. M3 needed to re-examine and provide a final reading for 3231/30 025 (10.76%) objects where the two reference microscopists did not agree (Figure 1).

Table 2 shows the distribution of egg types captured and identified by M1, M2 and M3. The majority of the objects identified in the images were *A. lumbricoides*–fertilized and negative (32 objects agreed per 100 objects identified each). Other egg type or microorganism was the least identified object (1 object agreed per 1000 objects).

**Table 2.** Frequency of final object readings, agreements between M1, M2 and M3 and variability scores per specimen type

Specimen type	Final object reading, n (%)	Agreement between M1 and M2, n (%)	Final object reading by M3, n (%)	Agreement between M1 and M3, n (%)	Agreement between M2 and M3, n (%)	Variability score	
						M1 and M2	M1, M2 and M3
Negative	9379 (31.24)	8184 (27.26)	1195 (3.98)	214 (6.62)	967 (29.93)	0.241	0.028
*Ascaris lumbricoides*–fertilized	9543 (31.78)	8811 (29.35)	732 (2.44)	520 (16.09)	67 (2.07)	0.130	0.019
*Ascaris lumbricoides*–unfertilized	1988 (6.62)	1854 (6.17)	134 (0.45)	59 (1.83)	56 (1.73)	0.392	0.073
Hookworm	3442 (11.46)	3174 (10.57)	268 (0.89)	62 (1.92)	32 (0.99)	0.196	0.043
*Trichuris trichiura*	3770 (12.56)	3670 (12.22)	100 (0.33)	187 (5.79)	45 (1.39)	0.043	0.005
*Schistosoma japonicum*	1870 (6.23)	1084 (3.61)	786 (2.62)	304 (9.41)	366 (11.33)	0.487	0.101
Other egg type or microorganism	33 (0.11)	17 (0.06)	16 (0.05)	4 (0.12)	2 (0.06)	14.121	2.909
Total	30 025 (100)	26 794 (89.24)	3231 (10.76)	1350 (41.78)	1535 (47.51)	–	–

### Agreement among reference microscopists

In general, M1 and M2 agreed on almost 90% of all the object readings. There was very good agreement between reference microscopists M1 and M2 (κ=0.86). Their agreement was not due to chance and may be true agreement (p<0.001). Relatively greater agreement between the two microscopists was observed with negative readings (8 184/30 025;27.26%) and *A. lumbricoides*–fertilized’ (8811/30 025 [29.35%]). M1 and M2 agreed least on other types of eggs or microorganisms (17/30 025 [0.06%]) (Table 2).

M3 labelled 3231 objects. M1 and M3 agreed on 41.78% (1350/3231) of all the image readings labelled by M3. There was fair agreement between reference microscopists M1 and M3 (κ=0.30). Their agreement was not due to chance and may be true agreement (p<0.001). M2 agreed more frequently with M3 (1535/3231 [47.51%]) compared with M1. There was fair agreement between reference microscopists M2 and M3 (κ=0.28). Their agreement was not due to chance and may be true agreement (p<0.001). M2 and M3 agreed more frequently with negative readings (967/3231 [29.93%]) (Table 2).

### Variability among reference microscopists

As shown in Table 2, the highest variability score between M1 and M2 was observed on other egg type or microorganism (14.121) followed by *S. japonicum* (0.487). A similar pattern was observed for variability scores among M1, M2 and M3, where other egg type or microorganism had the highest score (2.909) followed by *S. japonicum* (0.101). There was a decrease in variability across all specimen types when read by three microscopists compared with being read by just two microscopists.

**Figure 2.**
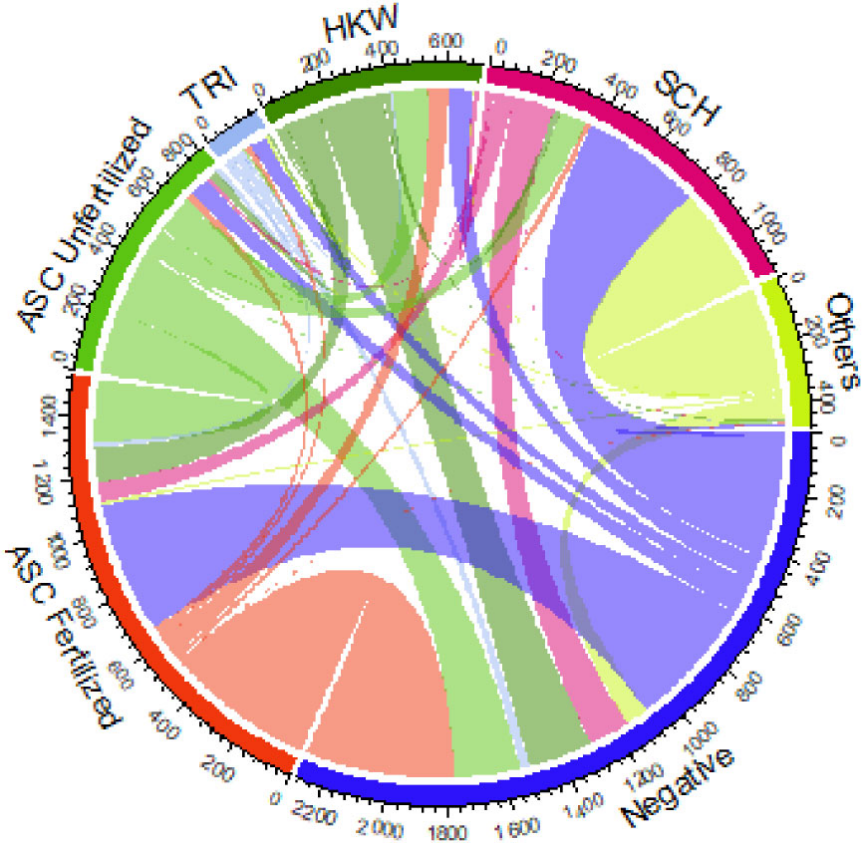
Chord diagram showing varying interpretations per label. Each node shows the frequency of the final label (ASC Fertilized: *A. lumbricoides*–fertilized; ASC Unfertilized: *A. lumbricoides*–unfertilized; TRI: *T. trichiura*; HWK: hookworm; SCH: *S. japonicum*; Others: other egg type or microorganism). Lines across nodes show relationships of disagreement by label. Thicker lines represent a greater frequency of disagreement from one label to another.

Of the 3231 objects labelled by M3, there were 346 (10.71%) objects where all three microscopists did not agree on the labels, accounting for 1.15% (346/30 025) of the total objects labelled. The distribution of disagreements across the objects of disagreement versus the final object readings can be found in Figure 2. The high variability score of other egg type or microorganism can be attributed to the number of *S. japonicum* labelled as other egg type or microorganism, as shown by the thick lines connecting their respective nodes. Among the target egg types, the higher variability score of *S. japonicum* can be attributed to it being labelled as negative or as other egg type or microorganism, as shown by the two thickest lines connecting *S. japonicum* to the respective nodes.

**Figure 3.**
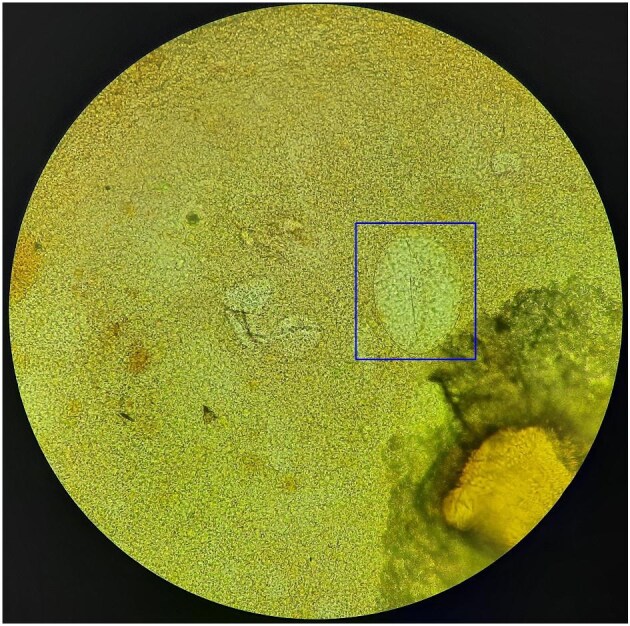
Boxed object where all three reference microscopists did not agree on the label. Objects were labelled as ‘other egg type or microorganism’, ‘negative’ and ‘*S. japonicum*’ by M1, M2 and M3, respectively. Observed under 400× magnification.

The most common label disagreements among all three microscopists were between negative, *S. japonicum* and other egg type or microorganism (65/346 [18.79%]). Examples can be found in Figure 3, where the first microscopist labelled the objects as other egg type or microorganism, the second microscopist labelled the objects as negative and the third microscopist labelled the objects as *S. japonicum* consistently across 65 objects.

## Discussion

Generally, high agreement was observed between the first two reference microscopists [26 794/30 025 [90%]), who had training and >10 y of experience in the field of medical parasitology. Similar results were shown between pathologist readings for histopathologic slides,^10^ where more experience in identification of microscopic objects provided overall high agreement.

The images contained the more common soil-transmitted helminth ova that are reliably diagnosed by microscopists, contributing to high agreement between the two reference microscopists.^16^ The microscopists generally agreed on fertilized *Ascaris* ova more than unfertilized ova. The most common mismatched pair for the first two microscopists was between fertilized *Ascaris* ova and negatives, consistent with findings from previous studies.^17^ Interestingly, six of the most common label disagreements among all three microscopists included negative and five of the top six mismatched label pairs include either fertilized or unfertilized *A. lumbricoides* ova. This may be due to varying morphologies of *Ascaris* ova, particularly decorticated eggs and the similarity to artifacts.^17^ Microscopists with varying levels of experience have previously been shown to present low agreement (33%) in parasite identification on the same samples.^12^ The data from this study suggest that some artifacts may look like ova and that some ova may look like artifacts, even among experienced reference microscopists.^17^ Fatigue may also act as an element that causes mislabelling, as the microscopists labelled approximately 250 objects of interest per hour in the span of 1 month and misclicking during the labelling process could have occurred. To mitigate potential fatigue and mislabelling, the expert microscopists were tasked with labelling the objects at their own pace; however, this cannot entirely rule out human error. A limitation of the study was the lack of intra-observer reliability testing, which may be considered in a future study.

In cases where the two reference microscopists disagreed on the reading, the reading of the third microscopist (M3) was used as the final reading. The third microscopist agreed with around 41% of M1’s readings and 47% of M2’s readings. From the perspective of having two of three microscopists agree on the label of an object, M3 resolved 88% of discrepancies between the first two microscopists. The κ statistic of M3 with M1 and M2 (0.30 and 0.28, respectively) was interpreted as fair agreement. McHugh^18^ provided a different interpretation that would classify the κ values (0.30 and 0.28) as minimal agreement, taking into consideration the reduction of agreement arising from the computation of the κ statistic, reducing agreement due to chance.

Objects can be confusing due to vague morphologies, which can be attributed to more than one parasite or possibly artifacts (Figure 3). Microscopists may have little experience with detecting *S. japonicum*, as the distribution of schistosomiasis is highly focal.^19^ This may contribute to confusion of *S. japonicum* ova with other parasite eggs or even artifacts by microscopists. To identify confusing parasite ova or objects, a consensus by two or more experienced reference microscopists is suggested to have a greater level of confidence in the reading of the object. Additionally, viewing other parasite eggs present on the same slide may increase the level of confidence in the reading of a particular object, as it gives the microscopist an idea of what the slide may actually contain.

There has been limited use of digital microscopy in the field of medical parasitology, which presents a different challenge for the reference microscopists. The static images used during the labelling process do not allow manipulation of focus and light intensity, hence there is a need to look for other similar ‘eggs’ or parasite material within the image to compensate for the inability to change focus. The advantage of using digital microscopy and static images is that the exact same field of view can be directly evaluated by different microscopists, showing the same amount of focus and resolution.

## Conclusions

In this study, three reference microscopists reliably provided a consensus on parasite ova identification in about 99.0% of the images observed. The high agreement of these reference microscopists shows that they serve a role in continuing capacity development in medical parasitology as teachers, trainers, referees and experts. Since the image set will be used to inform machine learning microscopy, greater confidence can be achieved with the results of the identified ova by AI, particularly in resource-limited settings.^20^ The availability of AI tools for the detection of parasite eggs may reduce referrals to other microscopists for commonly identified parasite ova since the AI will be trained to recognize images at the level of reference microscopists. Diagnosis can also become more standardized and objective by using this dataset to train AI in light of the lack of training and capacity in many settings. Referrals to medical parasitology specialists and reference microscopists can focus on the uncommon and confusing parasite ova seen in microscopy.

## Data Availability

The data underlying this article will be shared upon reasonable request to the corresponding authors. VYB should be contacted regarding the analyses in the manuscript and KAH should be contacted regarding image set inquiries.

## Co mpeting interest s

None declared.
